# “Nothing for Us Without Us”: A Mixed Methods Study Examining the Acceptability, Feasibility, and Impact of Involving Guardians of Children With Acute Lymphoblastic Leukemia in Tanzania as Public Contributors

**DOI:** 10.1002/cam4.71685

**Published:** 2026-03-02

**Authors:** Faraja Chiwanga, Guardian Advisory Board, Ruchius Philbert, David A. Richards, Abla Sami, Holly V. R. Sugg, Ida Österman Menander, Joanne Woodford, Louise von Essen

**Affiliations:** ^1^ Muhimbili National Hospital, Directorate of Medical Services Dar es Salaam United Republic of Tanzania; ^2^ Department of Women's and Children's Health, CIRCLE – Complex Intervention Research in Health and Care Uppsala University Uppsala Sweden; ^3^ Department of Paediatrics and Child Health Muhimbili National Hospital Dar es Salaam United Republic of Tanzania; ^4^ Department of Epidemiology and Biostatistics, College of Health and Social Sciences, School of Public Health Makerere University Makerere Uganda; ^5^ Department of Health and Caring Sciences Western Norway University of Applied Sciences Bergen Norway; ^6^ Department of Health and Care Professions, Academy of Nursing University of Exeter Exeter UK

**Keywords:** acute lymphoblastic leukemia, childhood cancer, guardians, mixed methods, public contribution, treatment adherence

## Abstract

**Introduction:**

In low‐ and middle‐income countries (LMICs) such as Tanzania, non‐adherence and treatment abandonment are major factors contributing to low childhood cancer survival rates. Providing guardians with reminders and information via an SMS intervention may increase adherence and reduce treatment abandonment. The purpose of the GuardiansCan project is to reduce guardians' abandonment of children's maintenance therapy for acute lymphoblastic lymphoma (ALL) in Tanzania, thereby increasing ALL survival rates in the country by developing and evaluating an SMS intervention. We report initial results from phase one of a mixed‐method examination of public contribution activities in the GuardiansCan project. We aimed to: recruit guardians of children treated for ALL to a Guardian Advisory Board (GAB) to contribute to the design and conduct of Study II within the GuardiansCan project (Study II) and the wider GuardiansCan project; and examine the acceptability, feasibility, and impact of GAB members' contribution from the perspective of GAB members and public contribution coordinators (coordinators).

**Methods:**

We adopted a convergent parallel mixed‐methods design, using impact logs and semi‐structured interviews, with data integrated at the point of analysis. During four workshops, GAB members provided suggestions and recommendations, which were recorded in impact logs. GAB members and coordinators were interviewed about the acceptability, feasibility, and impact of GAB members' contributions.

**Results:**

Nine guardians were recruited. GAB members made 63 suggestions and recommendations, of which 50 (79%) were implemented. Semi‐structured interviews resulted in seven categories: Meaning and value, Motivation and willingness to volunteer, Suggestions and areas for improvement, Barriers and challenges, Facilitators, Personal impact, and Research impact.

**Conclusion:**

Findings suggest GAB members' contribution was acceptable and feasible, and had an impact on both the research and GAB members themselves. Findings can inform how to meaningfully involve public contributors in LMICs.

## 1 Introduction

Projections from the 2022 Lancet Oncology Commission [1] indicate that by 2050, Africa will account for approximately 50% of the global childhood cancer burden. In 2019, the global net 5‐year childhood cancer survival rate was 37.4%, with significant regional variations, ranging from 8.1% in Eastern Africa to 83.0% in North America [2]. Protocolized treatments have led to substantial survival gains in high‐income countries (HICs) [3]. However, in many low‐ and middle‐income countries (LMICs), issues with infrastructure, care, and human resources make adherence to protocolized treatments challenging [4]. Non‐adherence is the extent to which patients do not follow protocols, for example, do not take medications as directed or attend clinic visits, ranging from occasional lapses to complete treatment abandonment [5]. If childhood cancer treatment is not adhered to or abandoned, the most likely outcome is death. To reduce abandonment, the Lancet Oncology Commission recommends increasing access to mobile Health (mHealth) interventions developed with local interest‐holders [1].

In sub‐Saharan Africa (SSA), guardians of children with cancer report unmet needs for information about the child's disease, condition, and treatment [6]. Barriers to adherence include lack of knowledge about cancer and its treatment [6, 7], fear of side effects, lack of belief in cancer curability [7], financial and transportation barriers [6], perceived (poor) health of the child [7], and thinking the child is cured because they look healthy [8]. Inadequate treatment information has been reported as a barrier to accessing chemotherapy in SSA [7]. Outside of SSA contexts, other reported barriers include child resistance [9], forgetfulness, and failing to link taking oral chemotherapy with recovery [10].

SMS text message‐based interventions (SMS interventions) have improved treatment adherence in SSA. Systematic reviews have shown mobile phone‐based reminders increase the uptake of early infant diagnosis of HIV [11] and one‐way SMS improves appointment attendance for a range of clinical conditions [12]. SMS interventions have been shown to increase HIV medication adherence for young people in Nigeria [13, 14], and mobile phone alarms are acceptable and feasible to improve treatment adherence among tuberculosis patients in Tanzania [15]. Therefore, an SMS intervention may help increase guardians' adherence to children's cancer treatment and reduce treatment abandonment.

We are undertaking the GuardiansCan project [16], with the overall purpose to reduce guardians' abandonment of children's maintenance therapy for acute lymphoblastic lymphoma (ALL) in Tanzania, thereby increasing ALL survival rates in the country by developing and evaluating an SMS intervention. Due to children being at home during ALL maintenance therapy (MT), treatment complexity, and responsibility placed on guardians as primary caregivers, there is a high risk of non‐adherence and abandonment. An SMS intervention, for e.g., providing treatment reminders and evidence‐based information, could help guardians adhere to ALL MT and reduce abandonment. To guide intervention development, we are following the UK Medical Research Council Complex Interventions Framework [17]. As part of the development phase [18], we will first conduct a qualitative study (Study II in the GuardiansCan project [16]) to explore needs and preferences (including barriers and facilitators for guardians adhering to children's ALL MT) and perceived barriers and facilitators regarding an SMS intervention helping guardians of children with ALL to adhere to their children's MT.

To align our research with guardians' needs and concerns, we established a Guardians Advisory Board (GAB) [19] to implement public contribution throughout the GuardiansCan project [16]. The approach helps uphold democratic principles, empowers individuals, bridges the gap between research and real‐world applications, makes research more relevant and impactful, and enhances the quality and ethical foundation of research [20]. Findings from HICs show public contribution can help formulate meaningful and relevant research questions, improve recruitment, develop clear and user‐friendly study materials [21, 22, 23], strengthen analysis and interpretation [24], and promote dissemination outside academia [25]. These impacts may be amplified in LMICs. Research design, conduct, and capacity building in LMICs have been dominated by the Neocolonialist model and Western epistemologies [26]. This influence may result in research failing to consider important cultural contextual factors [27], for example, local needs and preferences, and economic and sociocultural factors. Embedding public contribution in research may facilitate generating contextualized knowledge in LMICs [28].

### 1.1 Aims

We report results from phase one of a mixed‐methods examination of public contribution activities [19] in the GuardiansCan project [16], given recommendations for undertaking and reporting initial, midterm, and end‐of‐study public contribution evaluations [29, 30]. We will report end‐of‐study results upon project completion. Overall aims of the ongoing study [19] are to (1) involve guardians of children treated for ALL as GAB members in the managing and undertaking, analysis and interpretation, and dissemination phases of the GuardiansCan project [16] and (2) examine the acceptability, feasibility, and perceived impact of GAB members' contribution to the project from the perspective of the GAB members and public contribution coordinators (coordinators).

In phase one, we aimed to (1) recruit guardians of children treated for ALL to a GAB to contribute to the design and conduct of Study II within the GuardiansCan project [16] (Study II) and the GuardiansCan project and (2) examine the acceptability, feasibility, and impact of GAB members' contribution from the perspective of GAB members and coordinators. Objectives were to (1) examine the acceptability and feasibility of recruiting guardians to the GAB, (2) describe suggestions and recommendations made by GAB members during workshops, and the percentage implemented into the GAB, Study II, and the GuardiansCan project, (3) describe personal impacts on GAB members and coordinators recorded during workshops, (4) explore the acceptability, feasibility, and impact of GAB members' contribution from the perspective of GAB members and coordinators, and (5) describe the economic costs of GAB contribution.

## 2 Methods

### 2.1 Design

A convergent parallel mixed methods design [31] with data collected concurrently on the acceptability, feasibility, and impact of GAB members' contributions using impact logs and semi‐structured interviews. We transformed qualitative impact log data into quantitative data [32]. We analyzed datasets separately, giving quantitative (transformed impact logs) and qualitative (interviews) data equal priority. We integrated at the point of analysis by triangulating the datasets with quantitative recruitment data in a side‐by‐side comparison table [33, 34]. We explored the extent to which findings converged, diverged, complemented, or expanded upon each other [35].

### 2.2 Reporting

We followed Guidance for Reporting Involvement of Patients and the Public checklist (GRIPP2, see Appendix S1) [36] and the Standards for Reporting Qualitative Research checklist (SRQR, see Appendix S2) [37].

### 2.3 Public Contribution Framework

We used a study‐focused framework for involving GAB members [38]. Public contribution activities to inform Study II [16] included managing and undertaking the study (e.g., developing recruitment and retention strategies), co‐designing study materials, identifying data collection methods, and dissemination activities, including co‐writing a Kiswahili plain‐language abstract for the current study. The GAB continues to contribute to Study II (e.g., analyzing and disseminating results, co‐designing plain language summaries, and developing local dissemination strategies) and the next phases of the GuardiansCan project as planned for now [see ref. [16]].

### 2.4 Protocol Changes

We planned a GAB workshop to gain feedback on and interpret study findings [19]. We did not hold this workshop due to cost and time. We planned for an external interviewer fluent in Kiswahili to conduct interviews with coordinators; however, due to time limitations, interviews were conducted in English by a Swedish research team member.

### 2.5 GAB Members

Full eligibility criteria has been published elsewhere [19] and included being a guardian of a child discharged within the last 3–36 months from the pediatric oncology unit at Muhimbili National Hospital (MNH) (Dar es Salaam), after completing main treatment for ALL (i.e., induction treatment phase), ≥ 18 years old, Kiswahili‐speaking, primary school to postsecondary nontertiary education, and being a resident of one of the following regions: Dar es Salaam, Dodoma, Iringa, Lindi, Morogoro, Mtwara, or the island of Zanzibar. We excluded guardians with tertiary education, given that low education is associated with treatment abandonment [39].

#### 2.5.1 Recruitment of GAB Members

Coordinator FC conducted recruitment activities, supervised by Swedish research team members (LvE, JW) and in collaboration with the MNH (Dar es Salaam) pediatric oncology unit. In March/April 2024, we obtained a list of children diagnosed with ALL who had completed main treatment and were discharged from MNH (Dar es Salaam) pediatric oncology unit within the past 3–36 months (January 2021–December 2023) from the Office of Medical Records. We excluded children documented as deceased. We identified children residing in target regions and randomized their guardians using random.org to determine the call sequence. After the first call round, we used purposive sampling to inform call order, for example, based on gender and region not represented by guardians who had expressed interest.

Coordinator FC called guardians (June 2024) to explore inclusion criteria, explain the purpose, expected GAB composition (i.e., 6–8 members representing variation in gender, relationship to child, education level, and location), the reimbursement plan (i.e., travel and living costs; daily allowance equivalent to the government daily subsistence allowance for ISCED level 4 personnel), and explore initial interest and abilility to contribute. FC informed guardians that around 12 interested guardians would be invited to a recruitment meeting at MNH (Mloganzila) (≈2 h), after which a subset would be invited to the GAB. For guardians unreachable by phone, FC made up to five repeat phone calls.

At the recruitment meeting, coordinators FC and RP explained the purpose of the GAB, planned structure and frequency of workshops, and explored guardians' expectations and motivations for contribution. FC, RP, and PI LvE thereafter decided who to invite. Subsequently, FC and RP informed guardians whether they were invited to the GAB in individual meetings and provided invited GAB members with written information and a consent form. After providing consent, guardians completed a background questionnaire, e.g., gender, age, relationship status, relationship to the child with cancer, educational background, and location.

### 2.6 Setting

Tanzanian research team members (FC, RP) coordinated public contribution activities from MNH (Mloganzila). Coordinators were supported by a hospital administrative officer, who organized workshop logistics and reimbursements. LvE and JW provided FC and RP with training on public contribution during two face‐to‐face workshops in Tanzania and supervision via Zoom.

### 2.7 GAB Structure and Workshops

We held four face‐to‐face workshops to inform the design and conduct of Study II [16]. Coordinators maintained regular telephone contact with guardians, for example, to arrange transportation, e.g., boat travel for Guardians living on the islands of Zanzibar or Pemba, or bus transportation for those living in Mtwara and Lindi, and hotel accomodationin Dar es Salaam. We provided GAB members with name tags, branded notebooks, and carrying bags (see Appendix S3).

Workshop topics (Table 1) were discussed using activities including brainstorming, group discussions, feedback on written material, ranking procedures, and storytelling exercises.

**TABLE 1:** Guardians Advisory Board (GAB) workshop topics to inform the design and conduct of Study II within the GuardiansCan project (Study II).

Workshop	Main topic	Subtopics
1	Introduction to the GuardiansCan project and the GAB	Introduction to the GAB, the GuardiansCan project, and public contribution in research, and GAB members' expectations
		GAB structure (e.g., frequency, breaks, etc.) and location Communication plan for the GuardiansCan project and forthcoming results
		Any other business Information on topics to be covered in Workshop 2
2	Understanding and initial planning of Study II	Presentation and discussion of notes in Workshop 1 impact log and decisions made by the research team on discussed aspects Introduction to photovoice technique, FGD, and individual interview and discussion of the relevance of using these methods Recruitment of study participants, e.g., methods and procedures to use and regions to recruit from Background questions Frequency and setting of FGDs and whether GAB should facilitate FGDs Additional topics (i.e., topics added to the original Workshop 2 agenda after Workshop 1 by the research team informed by Workshop 1 discussions). Additional topics included the availability of mobile phones and motivation to participate in research Any other business Information on topics to be covered in Workshop 3
3	Further planning of Study II	Presentation and discussion of notes in Workshop 2 impact log and decisions made by the research team on discussed aspects Written information and consent forms Topic guides for FGDs Additional topics (i.e., topics added to the original Workshop 3 agenda after Workshop 2 by the research team informed by Workshop 2 discussions). Additional topics included exploring whether GAB members would be willing to try the photovoice technique in the future, asking if they would be interested in taking part in an upcoming Public Contribution training, and informing them about the planned involvement of HCPs in Study II, and asking for their input on questions to include in the FGDs with HCPs. Any other business Information on topics to be covered in Workshop 4
4	Final planning of Study II	Presentation and discussion of notes in Workshop 3 impact log and decisions made by the research team on discussed aspects Recruitment of HCPs to Study II Ethical considerations, such as privacy, identity protection, and use of sensitive information Additional topics (i.e., topics added to the original Workshop 4 agenda after Workshop 3 by the research team informed by Workshop 3 discussions). Additional topics included a second review of written information, consent forms, and topic guides for FGDs Any other business Discussion about logistics for individual interviews with GAB members and information on the next steps, for example, finalizing study II protocol and submission for ethical approval

Abbreviations: FGD, focus group discussion; GAB, Guardians Advisory Board; HCP, healthcare professional.

Each workshop lasted approximately 4 h. We provided lunch, regular breaks, and additional breaks (i.e., prayer time for Muslim GAB members). We held an additional workshop (attended by 4 female GAB members) to write the plain‐language summary in Kiswahili (see Appendix S4). This workshop lasted 2.5 h with an additional 1.5 h for breaks.

### 2.8 Data Collection

#### 2.8.1 Recruitment Data

We collected data on the number of potential GAB members identified, recruitment phone calls made, potential GAB members reached, eligible, recruited, and reasons for not attending the recruitment meeting.

#### 2.8.2 Impact Logs

In each workshop, coordinators reported in an impact log [40, 41] the date, who was involved, discussion (i.e., suggestions and recommendations), impacts on research (i.e., potential impacts on the project), impacts on guardians (i.e., personal impacts on GAB members and coordinators, and other comments). Coordinators presented each impact log at the start of the subsequent workshop to gather GAB members' feedback to ensure accuracy and support trustworthiness and the research team's decisions on whether suggestions and recommendations would be implemented. Coordinators audio‐recorded workshop discussions to ensure impact log accuracy.

#### 2.8.3 Semi‐Structured Interviews

An experienced interviewer external to the research team, fluent in Kiswahili and not involved in GAB workshops, interviewed GAB members, and IÖM interviewed coordinators to explore the acceptability, feasibility, and impact of GAB activities. Interview guides were informed by previous research [41, 42], and published elsewhere [19]. We conducted interviews 5–6 months after the GAB was formed.

### 2.9 Data Analysis

We analyzed the datasets separately before integrating quantitative recruitment data and (transformed) impact log data with qualitative interview data in an integrative mixed methods analysis.

#### 2.9.1 Recruitment

We used count data to report recruitment data.

#### 2.9.2 Impact Logs

FC and IÖM read, extracted, and summarized GAB suggestions and recommendations, alongside their rationales (where applicable), supervised by LvE. FC and IÖM categorized suggestions and recommendations as those made to inform: (1) the GAB, (2) Study II, and (3) the GuardiansCan project, which were reviewed by LvE. Next, JW categorized suggestions and recommendations across workshops. These were subsequently counted to transform the qualitative data into quantitative data [32]. The percentage of suggestions and recommendations implemented for each category and in total was calculated. IÖM extracted and summarized personal impacts on GAB members and coordinators, which were categorized and counted by AS.

#### 2.9.3 Semi‐Structured Interviews

Audio recordings were transcribed verbatim. The external Tanzania interviewer transcribed audio recordings of GAB members' interviews. FC checked transcripts against audio recordings, which were subsequently translated from Kiswahili into English by an external company (Semantix) using a Kenyan Swahili speaker. FC checked translation quality. An external company (CBG Konsult & Information AB) transcribed audio recordings of interviews with coordinators, and AS checked transcripts against audio recordings.

AS and FC individually coded and categorized English transcripts using manifest content analysis [43] and subsequently held a data analysis workshop to discuss and refine preliminary categories and subcategories. Next, we held a data analysis workshop with wider research team members (FC, LvE, AS, JW) to facilitate peer examination. We established trustworthiness [44] using disconfirming case analysis, peer examination, and keeping an audit trail to help maintain reflexivity and minimize bias.

#### 2.9.4 Mixed Methods Analysis

HVRS integrated quantitative recruitment data and (transformed) impact log data with qualitative interview data using a side‐by‐side comparison table [33, 34]. To triangulate datasets, HVRS identified key topics across datasets (recruitment, impact logs [research and personal impacts], semi‐structured interviews) and grouped them according to whether they related to the following areas: acceptability, feasibility, and impact on the GAB and/or impact on the research. In the side‐by‐side comparison table, each row represented a key topic, and each column represented a data source. The table was organized according to each area. Within each area, key topics were organized according to quantitative data on recruitment, the percentage of suggestions and recommendations implemented (research impacts), and/or the number of impacts on the GAB (personal impacts) recorded in impact logs, and qualitative data from interviews. HVRS, in discussion with JW and AS, mapped key findings from each data source to the table and explored where findings were concordant or in agreement with each other (convergence), offered different but non‐conflicting information on the same issue (complementarity), offered concordant but additional information on the same issue (expansion), appeared to be discordant or in disagreement with each other (divergence), or appeared in one dataset and not the other (silence) [35]. For each key topic, we recorded these meta‐inferences in the final table column.

### 2.10 Researcher Characteristics

FC is a female doctoral student (Master of Medicine in Internal Medicine, MSc in Endocrinology and Diabetes, and MSc in Public Health), physician at MNH, and a native Kiswahili speaker. LvE is a female Professor in Caring Sciences, a psychologist, and Principal Investigator for the project, specializing in mental health among people affected by somatic disease and has extensive experience in qualitative research, including studies involving public contribution. GAB members are nine guardians of children currently being treated for ALL in Tanzania. RP is a pediatric nurse at MNH, studying for an MSc in Public Health. DR is a male Professor in Mental Health Services Research and an experienced nurse, with extensive experience in qualitative research and public contribution. AS is a female research assistant (MSc in Community and Public Health). HVRS is a female Lecturer (Education and Research) in Health Services Research with extensive experience in qualitative and mixed methods research. JW is a female Associate Professor in Caring Sciences with extensive experience in qualitative research, including studies involving public contribution. IÖM is a female research assistant (MSc in Global Health).

We recognized our positionalities as Swedish and Tanzanian researchers may have influenced recruitment, analysis, and interpretation. To minimize the risk of imposing Western‐centric norms, public contribution activities were led by Tanzanian research team members FC and RP, with supervision from Swedish team members LVE and JW. Interviews with GAB members were conducted by a Tanzanian interviewer with experience in pediatric oncology research and knowledgeable of the context and cultural norms. Interviews were analyzed by FC and AS, who held joint analysis meetings to ensure cultural and contextual understanding and methodological rigor.

### 2.11 Ethical Considerations

We followed the Declaration of Helsinki. We obtained ethical approval from MNH (MNH/IRB/VOL.I/2023/080), the Tanzania National Health Research Ethics Review Committee (NIMR/HQ/R.8A/Vol.IX/4544), and the Swedish Ethical Review Authority (2023‐01381‐01). We collected informed consent from GAB members. GAB members had the right to withdraw from the GAB at any time without providing a reason. GAB members provided separate consent for interviews, and coordinators provided consent for interviews. We processed data following the Swedish Patient Data Act (2008:355), the General Data Protection Regulation (EU 2016/679), and the Tanzania National Institute for Medical Research (NIMR) research policy.

## 3 Results

### 3.1 Recruitment

**FIGURE 1:**
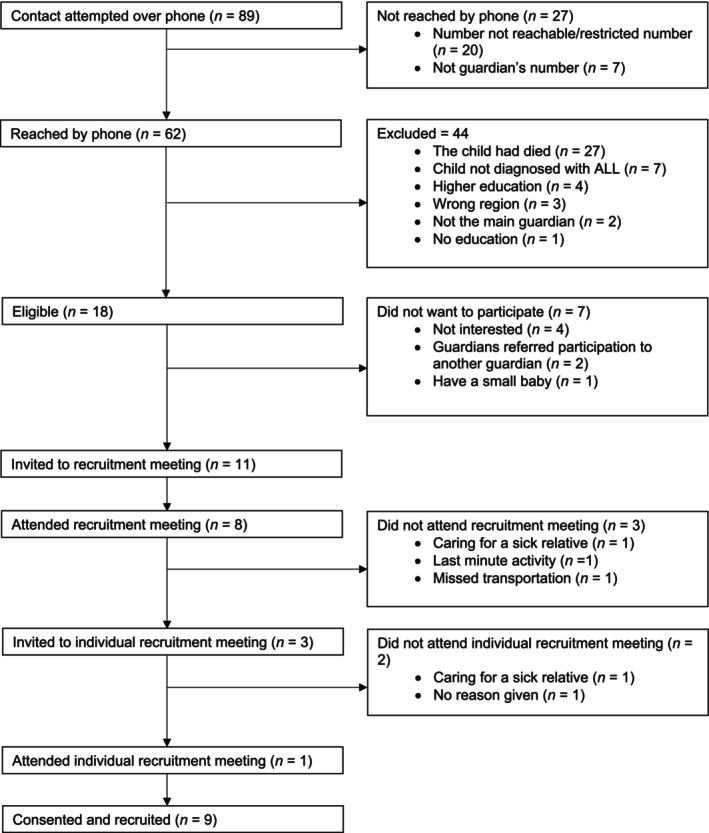
Recruitment of guardians to the Guardian Advisory Board.

Recruitment flow is summarized in Figure 1. Of the 89 potentially eligible guardians identified with phone contact attempted, 62 were reached, 18 were eligible, and 11 were invited to the recruitment workshop. Eight (7 women, 1 man) attended and were recruited. Three men who did not attend were invited to an individual recruitment meeting: one attended and was recruited. In total, nine guardians were recruited. We made 159 recruitment phone calls. On average, guardians responded after 1.5 call attempts (range 1–5). Of the 11 guardians invited to the recruitment workshop, six responded to the first call attempt, one to the second, three called back after one missed call, and one called back after two missed calls.

### 3.2 Characteristics of Guardian Advisory Board Members

Selected GAB member characteristics are reported in Table 2.

**TABLE 2:** Characteristics of Guardian Advisory Board (GAB) members.

	Gender	Age	Civil status	Relationship to child	Education level	Region
1	Woman	30–39	Married	Mother	Primary	Pwani
2	Woman	40–49	Divorced	Grandmother	Secondary	Dar es Salaam
3	Man	20–29	Married	Uncle—Maternal side	Primary	Lindi
4	Woman	30–39	Married	Mother	Certificate post‐secondary	Mtwara
5	Woman	30–39	Married	Mother	Secondary	Dar es Salaam
6	Woman	40–49	Married	Mother	Diploma post‐secondary	Unguja, Zanzibar
7	Woman	20–29	Single	Mother	Secondary	Dar es Salaam
8	Woman	40–49	Married	Mother	Primary	Dar esSalaam
9	Man	30–39	Married	Uncle—Maternal side	Secondary	Pemba, Zanzibar

*Note:* Age ranges are used to protect confidentiality.

### 3.3 Impact Logs

Categories of suggestions and recommendations made by the GAB to inform the GAB, Study II, and the GuardiansCan Project (research impacts), alongside examples and numbers implemented, are reported in Table 3. Overall, GAB members made 63 suggestions and recommendations, of which 50 (79%) were implemented. Suggestions and recommendations are reported in Appendix S5, alongside rationales for the suggestions or for non‐implementation.

**TABLE 3:** Research impacts on the GAB, Study II within the project (Study II), and the wider GuardiansCan project recorded in impact logs.

Category	Category description	Example suggestion and recommendation	*N*	*n* (%) implemented
Research impacts on the GAB				
Recruitment of GAB members	How to recruit additional men to the GAB	Recruit study participants via GAB members by calling guardians that they know (not implemented)	3	0 (0%)
Communication with GAB members	How the public contribution coordinators communicate with GAB members in between workshops	Contact GAB members 2 weeks in advance to remind them about the next workshop, and again 5 days before the workshop (implemented)	3	3 (100%)
Structure of the GAB	How the GAB should be structured, including when and where to meet, room facilities, and whether to invite partners	Workshops should not overlap with days guardians visit the pediatric oncology clinic (implemented)	10	10 (100%)
Other topics	Suggestion to provide GAB members with GuardiansCan project t‐shirts	GAB members should have project t‐shirts (implemented)	1	1 (100%)
Total			17	14 (82%)
Research impacts on Study II				
Method to collect data	Photovoice technique, FGD, or individual interview	Use FGDs in Study II (implemented)	1	1 (100%)
Recruitment of guardians	How to recruit guardians, including who to make recruitment calls, time and frequency of recruitment calls, and whether to use SMS	Ensure that recruitment from Zanzibar considers the island's five regions: North Unguja, South Unguja, Urban West Unguja, South Pemba, and North Pemba (implemented)	15	10 (67%)
FGDs	Logistics and set‐up of FGDs, including the structure, frequency, location, and facilitation of FGDs	Hold FGDs for between one and 2 h (not implemented)	7	6 (86%)
Data collection tools	Background questionnaires and topic guides for FGDs	No need to ask questions about religion, tribe, or type of phone (implemented)	8	8 (100%)
Participant facing materials	Information sheets and consent forms	No need to add to or amend the consent form (implemented)	4	4 (100%)
Ethical considerations	Harms/benefits, confidentiality	Include that a possible risk may be arriving at Dar es Saleem at night. Mitigate this risk with good communication with the research team about arriving time and assigning a driver to collect guardians (implemented)	4	4 (100%)
Total			39	33 (85%)
Research impacts on the wider GuardiansCan project				
Intervention	Mode of delivery of the mHealth intervention to be developed and evaluated in the GuardiansCan project, for example, Smartphone or Dumbphone	Develop a mHealth intervention that does not rely on smartphones (implemented)	7	3 (43%)
Total			7	3 (43%)
Total			63	50 (79%)

*Note:* Categories of suggestions and recommendations made by the Guardian Advisory Board (GAB) alongside examples, numbers implemented into the GAB, Study II within the project (Study II), and the wider GuardiansCan project are reported.

Abbreviations: FGD, focus group discussion; GAB, Guardians Advisory Board.

In total, 31 personal impacts on GAB members were recorded and categorized (see Table 4). No personal impacts on coordinators were recorded.

**TABLE 4:** Personal impacts on Guardian Advisory Board (GAB) members recorded in impact logs.

Category	Category description	Example personal impact	Number
Confidence and empowerment	Increased self‐confidence, sense of recognition, and belief in their own abilities	Appreciating being able to speak in front of people with higher education than one's own, which gave confidence	10
Personal motives	Motivation to support others, share knowledge, and be part of the solution, e.g., to reduce guardians' abandonment of children's maintenance therapy for ALL	Advising one another on how to support other guardians in taking care of their child	6
Motivation for caregiving	Growing role in cancer treatment decisions, e.g., ability to advocate for proper care, and increased adherence to their child's treatment	Restarting cancer treatment after previously giving up	5
Building relationships and community engagement	Appreciation of meeting others, sharing experiences, and supporting one another	Finding friends who can offer ideas on caring for the child	4
Knowledge acquisition	Increased understanding of ALL and GAB members' roles in the GAB/research	Understanding the ethical principles of the research	4
Cultural reflection	Reflections on caregiving roles based on cultural norms	Sharing that fathers may not count their child diagnosed with cancer as alive	2
Total			31

Abbreviations: ALL, acute lymphoblastic leukemia; GAB, Guardians Advisory Board.

### 3.4 Semi‐Structured Interviews

All GAB members (*n* = 4 face‐to‐face, *n* = 5 over the phone) and coordinators (*n* = 1 face‐to‐face and *n* = 1 via Zoom) were interviewed. Table 5 provides an overview of categories and subcategories derived from interview data for each area (acceptability, feasibility, and impact) explored. Supporting quotations, alongside ID number and gender, are provided for GAB members. Gender is not reported for coordinators as confidentiality would be compromised. We use the term “project” to refer to the GAB, Study II, and wider GuardiansCan project.

**TABLE 5:** Categories and subcategories derived from interview data related to each area (acceptability, feasibility, and impact) explored.

Area	Categories	Category definition	Subcategories
Acceptability	Meaning and value	Descriptions related to GAB members finding their contribution meaningful, valued, and aligned with their expectations	Positive experiences and gratitude
			Support for public contribution in research
	Motivation and willingness to volunteer	Descriptions of GAB members' motives and personal choices that led them to engage with the GAB	Voluntary nature of contribution
			Personal motives
	Suggestions and areas of improvement	Descriptions of GAB members' suggestions made to improve the GAB	
Feasibility	Barriers and challenges	GAB members' and coordinators' descriptions of experienced barriers and challenges	Anxiety and fear
			Knowledge and understanding
			Travel and communication
	Facilitators	GAB members' and coordinators' descriptions of factors that facilitated meaningful contribution	Coordinator training and mentorship
			Group support
			Logistics and organization
			Supportive and meaningful engagement
Impact	Personal impact	GAB members' and coordinators' descriptions of how they had been impacted on a personal level	Building relationships
			Confidence and empowerment
			Learning and knowledge acquisition
			Motivation for caregiving
	Research impact	GAB members and coordinators descriptions of how GAB members contributed to the design of the project through their lived experience	

#### 3.4.1 Acceptability

We generated three categories: *Meaning and value* (two subcategories), *Motivation and willingness to volunteer* (two subcategories), and *Suggestions and areas of improvement*.

##### 3.4.1.1 Meaning and Value

###### 3.4.1.1.1 Positive Experiences and Gratitude

GAB members and coordinators described GAB members' contribution as positive, meaningful, fulfilling, valued, and even transformative. GAB members felt supported, expressed gratitude for the opportunity to contribute, and emphasized the importance of the initiative: “This initiative has been life‐changing; they have supported us in ways that are difficult to put into words.” (GAB member 6, woman).

GAB members expressed a strong sense of pride in being recruited: “I felt honored to be a part of it.” (GAB member 4, woman). Coordinators also observed these positive experiences, and recognized GAB members' enthusiasm and strong engagement, for example: “Sometimes they call to ask about the next activity. They will ask ‘when is the next workshop?’” (Coordinator 2).

###### 3.4.1.1.2 Support for Public Contribution in Research

GAB members expressed support for the idea that individuals with lived experience, particularly caregivers, should play a role in shaping research, to ensure grounding in real‐world experiences:

If they [research team] had only consulted people based on their [academic] level of education, they might have gained a lot academically, but they would have missed out on many insights from real‐life challenges faced by those directly affected. (GAB member 9, man)

Others emphasized public contribution can build stronger, more trustworthy research and should be embraced despite any perceived challenges: “I would also like to advise other researchers not to be afraid of collaborating with the community. It may seem costly or challenging, but in the end, you gain reliable and valuable information.” (GAB member 7, woman). This view was supported by coordinators, who voiced the importance of involving the public throughout the research cycle to “get acceptable and implementable solutions for challenges or problems” (Coordinator 2).

##### 3.4.1.2 Motivation and Willingness to Volunteer

###### 3.4.1.2.1 Voluntary Nature of Contribution

GAB members highlighted the importance of joining willingly and understanding the purpose of the GAB: “I understood them [the coordinators], and I felt comfortable joining. They explained everything clearly to me, and after understanding, I willingly and voluntarily decided to join” (GAB member 9, man). As a coordinator explained, the recruitment process was planned to avoid any coercion by “not using somebody they know, who they are working with, so that they don't feel obliged to participate” (PC 1).

###### 3.4.1.2.2 Personal Motives

GAB members described a variety of personal motives for contributing, including a sense of responsibility as caregivers, a belief they were representing others in similar situations, an opportunity to support other guardians, a desire to learn, and the opportunity to gain valuable information:

I realized I could find comfort in understanding how these children live in such conditions. I also thought I could gain insights or knowledge that would help me in the future, especially in knowing how to care for my child, who also has blood cancer. Through this project, I knew I would gain valuable information and different ways to navigate this situation. (GAB member 1, woman)

##### 3.4.1.3 Suggestions and Areas of Improvement

Although coordinators and some GAB members had no suggestions for improvement, indicating satisfaction with the GAB, some GAB members recommended including more guardians from diverse backgrounds and different caregiving experiences:

What I face with blood cancer may not be the same as someone else's experience. So, if improvements are being made, they should also take into account conditions like skin cancer and kidney cancer—what challenges do they [guardians] face? (GAB member 5, woman)

Other members highlighted the importance of increasing geographic diversity: *“*There are certain regions where we feel membership could be added. For example, we talked about including more regions like Tanga.” (GAB member 9, man).

In addition, whilst some GAB members requested higher compensation, others proposed adopting more flexible communication methods to improve accessibility, particularly for those with limited resources: *“*We should be able to hold [GAB] meetings using various methods. Those with smartphones should be able to participate via phone… We also suggested that board members be provided with smartphones because some do not have phones” (GAB member 4, woman).

#### 3.4.2 Feasibility

We generated two categories: *Barriers and challenges* (three subcategories) and *Facilitators* (four subcategories).

##### 3.4.2.1 Barriers and Challenges

###### 3.4.2.1.1 Anxiety and Fear

Some GAB members expressed initial hesitance, anxiety and fear, and safety concerns, at times linked to recent events: “At that time, there were reports of organ trafficking circulating online, like kidney theft and other cases. This only increased our fear” (GAB member 3, man). Some female GAB members described concerns raised by spouses and family members, reflecting the influence of sociocultural norms and gender roles on household decision‐making: “When I was selected for the advisory board, I was ready and willing to take on the role. However, since I am a married woman, when I told my husband about it, he didn't easily accept it.” (GAB member 4, female). However, one disconfirming case described family support: *“*I didn't face any challenges or difficulties because I embraced this and discussed it at home.” (GAB member 6, woman).

While GAB members viewed phone‐based recruitment as practical and efficient, being contacted without prior context or personal connection made it difficult to build trust and understand the purpose of the GAB: “I kept wondering, ‘How does this person know me? I have a child with cancer—could they really be at Muhimbili [hospital]?’” (GAB member 8, woman). For some, being invited to meet unfamiliar people in a new place, with limited information, caused initial unease: “People were nervous going to meet someone you don't know, sleeping in a place you've never been to, and not knowing who you'll be interacting with.” (GAB member 1, woman).

###### 3.4.2.1.2 Knowledge and Understanding

Although GAB members did not report challenges in this category, coordinators expressed concerns related to GAB members' understanding of the project, overall research principles, and their role, alongside worries about their “*lack of confidence*” (PC 2) and unfamiliarity with research methods: “Of course, they can come up with suggestions that are not good for science. Again, you need to balance that.” (PC 1). However, coordinators also mentioned that, with support and repeated engagement, GAB members became more confident, gained knowledge, and developed a clearer understanding:

I was not sure if we were going to succeed in getting what we thought we were going to get. I think the second workshop was the point where they understood their role. And once they did, everything went very smoothly. (PC 1)

###### 3.4.2.1.3 Travel and Communication

Coordinators reported challenges with travel for those living in remote areas, requiring long journeys to attend meetings:

They need to have an overnight sleep somewhere [on transit]. Initially, we didn't know that, so they were coming straight from their hometowns. But then they would arrive at 2 am, and in the morning they have to go to the workshop, and they get so tired. (PC 1)

Additionally, inconsistent phone connectivity in certain regions was a barrier: “You call them at one time, they are not reachable… Because they are in a place where there is no connectivity.” (PC1). However, GAB members did not raise similar concerns, with logistical arrangements considered to be handled in advance and with care: “When I leave home, I plan accordingly and feel safe travelling to and from the workshops. I also know that transportation is available” (GAB member 6, woman).

##### 3.4.2.2 Facilitators

###### 3.4.2.2.1 Coordinator Training and Mentorship

Coordinators described being taught about public contribution, which facilitated their understanding of the GAB's purpose and how to work with its members. Training made them feel confident in leading and coordinating activities, and they appreciated the research team's support and guidance. “I completed the training [on public contribution]. And I thank the research team for that opportunity. So, that training also helped me a lot to lead, or to coordinate, this study” (PC 2).

###### 3.4.2.2.2 Group Support

Support and encouragement from one another, along with a collective sense of purpose within the GAB, served as powerful facilitators. Being part of an initiative that benefited families facing similar challenges, with a shared commitment and enthusiasm, was important for continued active contribution: “As parents, we found comfort in being there, seeing each other, greeting one another, and meeting in person. We have become like family” (GAB member 6, woman).

###### 3.4.2.2.3 Supportive and Meaningful Engagement

GAB members found the environment supportive, emphasizing the importance of being engaged in a way that made them feel valued, heard, and connected to the project: “The way they conducted themselves was so encouraging that I never wanted to miss any upcoming workshops” (GAB member 7, woman). Open, non‐judgemental and transparent communication and feedback facilitated this: “I felt the process was very transparent—whenever we discussed something, we received feedback, and we could see that the suggestions we made were considered and implemented accordingly” (GAB member 7, woman). By acknowledging GAB input and visibly integrating it into project decisions, GAB members felt empowered and their contribution was reinforced: “After we shared our opinions, they accepted them, adjusted their approach, and even praised us for being prepared and offering good advice.” (GAB member 1, woman).

GAB members also emphasized the importance of equal treatment, regardless of educational or social background, to help dismantle perceived hierarchies and promote genuine engagement:

[Coordinator] treated us as equals—they didn't look at our education levels or anything like that. When you sit with [coordinator], you feel like you are on the same level, like they understand your position and can walk in your shoes. [Coordinator] didn't create a hierarchy. They helped me a lot in this research because they never acted superior. (GAB member 5, woman)

###### 3.4.2.2.4 Logistics and Organization

Practical and consistent logistical support facilitated GAB contribution, including timely communication, clear meeting schedules, and reliable transportation arrangements:

If I needed to attend a workshop but didn't have the money for transportation, the project provided support, including transportation. That motivated me to participate fully because I had no concerns about transport or meals. So, I appreciated the support I received. (GAB member 2, woman)

Coordinators also recognized the importance of providing GAB members with the necessary tools for contribution and to help them feel valued, for example: “We give them branded bags. We give them branded notebooks. We give them (name) tags. So, they come like they are going to a proper conference” (PC1).

#### 3.4.3 Impact

##### 3.4.3.1 Personal Impact

We generated two categories: *Personal impact* (four subcategories) and *Research impact*.

###### 3.4.3.1.1 Building Relationships

Meaningful, trust‐based relationships were developed among members and coordinators. GAB members described building a social network and collective group identity:

When one of us is going through something, another offers advice on what to do. We've truly become like a family, and as a family, we share so much. Even beyond discussions about illness, we have built a strong bond—and that, too, is a great benefit we have received. (GAB member 5, woman)

Building relationships with other guardians reduced feelings of isolation and fear, fostered hope, and provided guardians with an opportunity to share caregiving ideas: “I've found some comfort, in knowing that I am not alone … there was a time I felt hopeless, but now I see hope at the end of the tunnel” (GAB member 7, woman). GAB members valued connecting with different professionals in a collaborative and supportive environment: *“*Another benefit of being part of this initiative is the sense of unity. We interact with different people, such as doctors, nurses, and other healthcare professionals” (GAB member 6, woman). These relationships created a sense of community and mutual support extending beyond the GAB's formal purpose.

###### 3.4.3.1.2 Confidence and Empowerment

Over time, GAB members experienced increased self‐confidence and empowerment, particularly in relation to speaking during workshops:

The greatest benefit I've gained is self‐confidence—the realization that I can stand in front of people and speak about something. I considered myself just an ordinary person, and I thought that if I shared my ideas elsewhere, they might not be valued. But today, I am speaking in front of doctors and international guests, and my thoughts are being respected. (GAB member 7, woman)

###### 3.4.3.1.3 Learning and Knowledge Acquisition

GAB members reported deepening their knowledge, particularly about caring for their own children: “I learned a lot. First, I gained experience and understood how to access services more easily. I also realized the importance of psychological support when facing challenges” (GAB member 2, woman). This learning also gave GAB members a sense of hope: “We need to guide them [the child with cancer], just as we would guide any other child without this illness. That way, even though my child has this condition, they too can have hope for life.” (GAB member 1, woman). GAB members also reported learning more about research and developing communication and discussion skills: “We learned a lot while creating the consent forms, including the importance of voluntary participation. We also learned to engage in discussions, further develop this project, and encourage our peers” (GAB member 2, woman). The anticipation of learning something new was expressed as motivating continued contribution: “I would be eager to know what new topic would come up next time. That's why, when the meeting day arrived, I always made sure to attend” (GAB member 1, woman).

Coordinators described growth in their own knowledge and research skills in public contribution: “Being one of the coordinators has helped me a lot to put the theoretical knowledge gained in the training into practice” (PC 2). Coordinators also reported developing a better understanding of guardians: “But another benefit, I have received working with the GAB members to understand their needs or requirements, and the challenges they are going through” (PC 2).

###### 3.4.3.1.4 Motivation for Caregiving

Contribution provided GAB members with a sense of purpose, responsibility, and fulfillment associated with their caregiving roles. For some, contribution reinstigated care, such as resuming taking their child to follow‐up visits: “This board has awakened something in guardians who had given up and left their children without treatment. When awareness is raised, a guardian finds themselves taking the child for treatment. This is a very significant contribution” (GAB member 5, woman). For others, it strengthened their commitment to their child's care: “I have gained so much from these meetings, even in caring for my child … I have never missed a single appointment for my child [since contributing to the GAB]—not even a single dose of medication or any treatment.” (GAB member 4, woman).

##### 3.4.3.2 Research Impact

In shaping research, GAB members provided a deeper understanding of contextual and cultural considerations, including challenges not previously considered or described in the literature: “So, it's that understanding that maybe, when you plan, that you don't get in the literature, you cannot review in the literature” (PC 1). GAB members provided crucial guidance on culturally sensitive communication strategies, including the emotional impact of language and question phrasing:

When asking a parent a question, the first question should be about the child's well‐being. If the child is no longer present, and you start asking about them, it could be painful for the parent. So, we designed questions to ensure we approach the topic sensitively. (GAB member 5, woman)

GAB members and coordinators reflected on the impact of their contribution on the design of Study II, including shaping the study protocol, using understandable and inclusive language in study materials, raising ethical considerations, and informing focus group topic guides, for example: “They shared their suggestions on different questions mentioned in the interview guides … they helped a lot with making sure that the questions in the interview guides are very clear.” (PC 2). GAB members also helped identify potential challenges and practical solutions, for example, related to recruitment strategies: “I also suggested that for numbers that don't ring at all, we should increase the number of attempts significantly and call at different times” (GAB member 9, man).

### 3.5 Mixed Methods Analysis

The side‐by‐side comparison table (Table 6) provides finding summaries from each data source (recruitment, impact log data [research and personal impacts], interviews) and meta‐inferences developed through data triangulation for each key topic (*n* = 15) identified across datasets. Key topics are grouped into the following areas: acceptability, feasibility, and impact on the GAB and/or the research. We summarized the data for each type of meta‐inference (convergence, *n* = 2; expansion, *n* = 7; complementarity, *n* = 3; silence, *n* = 3) below.

**TABLE 6:** Side‐by‐side comparison table showing summaries of findings from each data source for each key topic and related meta‐inferences.

Area	Key topic	Recruitment	Summary of impact log data (research and personal impacts^a^)	Summary of semi‐structured interview findings	Meta‐inference
Acceptability and feasibility	Practicalities and structure of the GAB	N/A	100% (*n* = 10) of suggestions and recommendations on how the GAB should be structured (i.e., when and where to meet, room facilities, and whether to invite partners) implemented; suggestion to provide GAB members with project T‐shirts implemented	Whilst coordinators suggested challenges related to travel and communication, GAB members did not (largely due to the practical support already provided). Compensation was acceptable, with only some wanting an increase. Coordinators noted the importance of providing members with necessary tools to participate, and members suggested more flexible communication methods, e.g., smartphones; diverse meeting options to facilitate accessibility	Complementarity: the quantitative and qualitative datasets provided different suggestions for improvement
	Support of and communication with GAB members	N/A	100% (*n* = 3) of suggestions and recommendations on communication with GAB members implemented	Support from one another and a collective sense of purpose within the GAB served as powerful facilitators. Despite some initial feelings of anxiety and fear, GAB members felt supported due to transparent, open and non‐judgmental communication from coordinators. They emphasized the importance of feeling valued, heard, and connected to the project; and of being treated equally, regardless of background	Expansion: whilst deemed acceptable in the qualitative data, quantitative suggestions for improvement were made and implemented
	Recruitment to and composition of the GAB	159 phone calls; 18 of 62 guardians phoned eligible; 7 females/2 males recruited	0% of suggestions and recommendations on how to recruit additional GAB members implemented (i.e., via existing members and local clinics) implemented due to risk of selection bias	GAB members emphasized the importance of joining the GAB willingly and understanding the purpose of the GAB. While members felt phone‐based recruitment was practical and efficient, it made it difficult to build trust and understand this purpose. Members recommended including more guardians from diverse backgrounds, with different caregiving experiences, and from more diverse geographical locations	Expansion: qualitative data offered broader suggestions for addressing recruitment issues highlighted in recruitment information
Acceptability and impact on GAB members	Motivations to take part in the GAB	N/A	6 reported personal impacts including motivations to support others, share knowledge, and be part of the solution	GAB members expressed various personal motives for taking part, including a sense of responsibility as caregivers, belief in representing others in similar situations, and the opportunity to gain and share information and improve understanding for themselves and others	Complementarity: original motives discussed in qualitative data versus impact on motives in quantitative data
	Feelings towards taking part in the GAB	N/A	Not reported	Both GAB members and coordinators described members' contribution as positive, meaningful, fulfilling, and valued. It gave members a sense of honor and pride. Members expressed gratitude for the opportunity to contribute, showed enthusiasm and strong engagement, and emphasized the importance of individuals with lived experience playing a role in shaping research	Silence in quantitative data
Impact on GAB members	Confidence and empowerment	N/A	10 reported personal impacts including Increased self‐confidence, sense of recognition, and belief in their own abilities	Over time, GAB members experienced increased self‐confidence and empowerment, for example, they increasingly felt comfortable speaking during workshops and sharing their thoughts. Coordinators noted GAB members' confidence improving with support and repeated engagement	Expansion: qualitative data added deeper insights into the personal impact of GAB participation including reasons for impact and members' feelings about impact; coordinators' reports converged with and expanded upon the quantitative data by adding their perspectives
	Motivation for caregiving	N/A	5 reported personal impacts focused on advocating for proper care and increased treatment adherence	Contribution provided GAB members with a sense of purpose, responsibility, and fulfillment associated with their caregiving roles which strengthened their commitment to their child's care	
	Learning and knowledge acquisition	N/A	4 reported personal impacts on knowledge focused on increased understanding of acute lymphoblastic lymphoma and GAB members' roles in the GAB and research	GAB members reported how workshops deepened their knowledge, particularly about caring for their own children, which gave them hope for the future. They also discussed learning more about research and developing communication and discussion skills, and coordinators noted such understanding improving with support and repeated engagement	
	Building relationships and community engagement	N/A	4 reported personal impacts including appreciation of meeting others, sharing experiences, and supporting one another	GAB contribution resulted in meaningful, trust‐based relationships among members and coordinators. Members described how a social network and collective identity were built, which reduced feelings of isolation and fear, fostered hope, and enabled sharing of caregiving ideas	
Impact on research	Overall impact on the research	N/A	78% (*n* = 36/46) of suggestions and recommendations for Study II or the intervention were implemented	Overall, GAB members and coordinators noted the impact of members' contributions on Study II. They acknowledged members enhanced the overall quality and relevance of the research by providing different and unique perspectives, offering cultural insights not previously considered or described in the literature, contributing to a better understanding of their needs and context, and sharing valuable knowledge and experiences	Complementarity: quantitative data captured practical suggestions; qualitative data captured how and why members contributed
	Materials	N/A	100% (*n* = 8) of suggestions and recommendations for background questionnaires and focus group topic guides implemented. 100% (*n* = 4) of suggestions and recommendations for information sheets and consent forms implemented	GAB members and coordinators noted impacts on questionnaires and topic guides, particularly in improving clarity and cultural appropriateness, and in using inclusive, understandable, and sensitive language	Convergence
	Ethical considerations	N/A	100% (*n* = 4) of suggestions and recommendations regarding harms/benefits and confidentiality were implemented	Both GAB members and coordinators emphasized members' contribution to identifying and addressing potential ethical concerns in the recruitment and data collection processes	Convergence
	Focus group discussions	N/A	Suggestion to use focus groups as a method to collect data implemented; 86% (*n* = 6/7) of suggestions and recommendations on focus group discussions logistics and set‐up implemented	Only topic guides mentioned (see above)	Silence in qualitative data
	Recruitment of guardians	N/A	67% (*n* = 10/15) of recruitment suggestions and recommendations implemented (e.g., to consider regional differences, and adapt the timing of phone calls)	GAB members and coordinators reflected on the significant contribution of members in identifying recruitment challenges and refining recruitment strategies, including the timing and frequency of phone calls, as well as communication strategies tailored to families' situations	Expansion: both datasets mentioned some overlapping and some unique contributions
	Intervention	N/A	43% (*n* = 3/7) of suggestions and recommendations implemented	Not discussed	Silence in qualitative data

Abbreviation: GAB, Guardians Advisory Board.

^a^ For research impacts, the percentage and number of suggestions and recommendations implemented into the GAB, Study II within the project (Study II), and the wider GuardiansCan project are reported. For personal impacts, only numbers are reported as the percentage implemented is not applicable.

Whilst convergence between datasets was apparent regarding the impact of GAB members' contributions on the research, e.g., “Materials” and “Ethical considerations,” for the majority of topics, datasets expanded upon each other. For example, in the topic “Support of and communication with members,” quantitative data expanded on qualitative data, suggesting support and communication was acceptable and also offered suggestions and recommendations for improvement. For the topic “Recruitment to and composition of the GAB,” qualitative data offered suggestions for addressing recruitment challenges beyond those provided in the quantitative data. For all topics within “Impact on GAB members,” qualitative data expanded on the quantitative data by providing deeper insights into how and why GAB contribution resulted in personal impacts, how members felt about these impacts, and how coordinators perceived them. In terms of impact on the research, datasets expanded upon each other by describing some overlapping and some unique contributions of members to “recruitment strategies.”

Complementarity information was offered by the datasets for three topics. To improve the “Practicalities and structure of the GAB,” quantitative and qualitative data provided different yet complementary suggestions. For “Motivations to take part in the GAB” in the qualitative data, original motives were described, whereas the personal impact of being a GAB member on these motivations was reported in the quantitative data. For “Overall impact on the research,” quantitative data showed the extent to which members' suggestions were implemented and qualitative data captured how and why members were able to impact the research.

Where there was silence in the quantitative data, qualitative data provided information on members' overall “Feelings towards taking part in the GAB,” including their views on the importance of public contribution, and coordinators' perceptions of their contribution. On the other hand, quantitative data demonstrated the impact of GAB members' suggestions regarding the “intervention” and “focus groups,” however the qualitative data was silent on these topics.

### 3.6 Economic Costs of GAB Contribution

The cost was approximately 10,746 USD. This cost included: transport (2687 USD), accommodation (1657 USD), meals (758 USD), reimbursement costs (e.g., daily allowance equivalent to the government daily subsistence allowance for ISCED level 4 personnel) (2109 USD), management cost (venue hire and administration support) (3108 USD), and stationery (427 USD).

## 4 Discussion

To our knowledge, this is the first study to examine the acceptability, feasibility, and impact of public contribution in health research in Tanzania. Findings from impact logs and interviews suggest public contribution activities had an impact on the research, with 79% of suggestions and recommendations implemented. Findings also revealed GAB members and coordinators found public contribution activities acceptable and feasible, resulting in personal impacts for GAB members, including building relationships, increasing confidence and empowerment, acquiring knowledge, and motivating caregiving.

Similar to other research [45], contribution impacted several important research design decisions. Of the suggestions and recommendations informing Study II, 85% (33/39) were implemented, similar to the 81% implementation rate reported in impact logs for an RCT of a digital intervention for schizophrenia‐spectrum psychosis [40]. Qualitative findings converged with and extended quantitative findings, with GAB members and coordinators considering GAB contribution to impact the research. For example, GAB members recommended using focus group discussions rather than photovoice techniques, changing the design of Study II [16]. Other research suggests public contribution can facilitate using more pragmatic study designs and raising considerations researchers would otherwise fail to anticipate [29]. The recommendation not to use photovoice in the GuardiansCan project was of particular interest given photovoice techniques are recommended with seldom‐heard and marginalized populations and are used in both HIC [46, 47] and LMIC settings, including Tanzania [48, 49]. We originally suggested using photovoice techniques to facilitate guardians' verbal expression of emotions and experiences [50], which may suggest we held incorrect assumptions about the population's ability to verbalize their emotions and experiences. This may have led to using an uneccessary, or even unacceptable, study design. Failure to embed public contribution in African research settings has been suggested to risk using inappropriate research protocols for the local context [51].

A further impact on the research from quantitative data concerned the type of mHealth intervention to be developed in the GuardiansCan project. Initially, we proposed exploring several mHealth delivery options (see ref. [16]). However, GAB members recommended, due to minimal smartphone, the only feasible solution would be a dumbphone intervention, for example, an SMS intervention. Interestingly, there was silence in interview data regarding this impact. However, interview findings provided complementary information to impact log findings regarding the overall impact on the research, with coordinators describing how GAB members raised cultural considerations not previously considered or described in the literature. Such impacts underscore the importance of embedding public contribution into research in LMICs to facilitate generating contextualized knowledge [28] and ensure local needs and preferences are recognized and prioritized.

Building trust with GAB members was a key facilitator for public contribution activities, as recorded in impact logs and expanded in interview data consistent with previous research [52]. GAB members described initial anxiety, fear, and hesitation when approached to join the GAB, shaped by contextual factors, that is, reports of organ trafficking and sociocultural norms and gender roles shaping household decision‐making. Building trust may be particularly important when working with underrepresented or seldom‐heard groups [53] Qualitative findings suggested open communication, feedback, and mutual respect were essential facilitators for building trust. Other research [54] has identified insufficient and poor communication with public contributors, for example, regarding how suggestions and recommendations are used, is a barrier to effective public contribution. Wider literature also suggests the importance of involving public contributors in decision‐making and ensuring regular feedback and transparent communication [55]. Our qualitative findings also highlighted the importance of treating GAB members equally to dismantle perceived hierarchies between contributors and coordinators. Equitable power sharing between public contributors and research team members has been identified as a facilitator to public contribution in the wider literature [56]. We designed public contribution activities to be led by local researchers in Tanzania (i.e., coordinators) to facilitate a deeper contexual understanding and trust [19], which may explain how initial hesitancy was successfully overcome.

Several personal impacts were reported in impact logs and expanded on in interviews. Whilst focus on identifying the impact of public contribution on research is growing, less is known about the personal impact on those involved, e.g., public contributors and the research team [52]. Identifying personal impacts is especially important, given public contribution is justified both by its potential to improve research and its recognition as an important human right [57], especially when involving seldom‐heard groups and underserved communities [55]. One important personal impact reported by GAB members in impact logs and expanded upon in interviews was the benefit of social support from others in a similar situation. GAB members also valued building relationships with the coordinators. Similar benefits have been reported elsewhere [52], highlighting the importance of developing long‐term relationships to successfully embed public contribution into research.

Another significant personal impact reported by guardians was increased adherence to their child's cancer treatment. Findings from impact logs and interviews highlighted one GAB member resumed cancer treatment after stopping treatment, and others became more motivated to adhere to their child's treatment. Similar findings have been reported elsewhere, including increased knowledge about their health condition [52] and improved medication adherence [54]. Increased adherence may have long‐term impacts on the health of children cared for by GAB members.

Our findings suggest compensation provided to GAB members was acceptable, with only some suggesting an increase. There is wider debate concerning how public contributors should be compensated to address economic disparities, especially when advisory boards include both public and professional members [58]. GAB members mentioned other compensatory facilitators, e.g., providing smartphones to help sustain communication and facilitate contribution without needing to travel long distances. However, our findings suggest providing travel and living costs, and an allowance equivalent to the government's daily subsistence allowance for ISCED level 4 personnel to guardians in Tanzania may be acceptable. The total cost of public contribution activities was 10,746 USD (excluding coordinators' costs). To our knowledge, public contribution research in Tanzania [59] and other SSA countries [28, 60, 61] has not reported the cost of contribution activities. In a review of public contribution in cancer research, only one‐third of papers provided any description of costs/remuneration [62]. Comparing costs with similar activities is therefore not possible. As research in LMICs often has lower budgets than in HICs [61], whether our costs are realistic or sustainable in similar LMIC contexts is a question warranting further investigation. If public contribution is to be widely embedded in Tanzania, there is a need for national‐level guidance on financial compensation and wider budget considerations to be co‐developed with public contributors to inform sustainable public contribution in the setting.

Interestingly, while coordinators raised logistical barriers in interviews, GAB members did not. This suggests the time and resources invested in coordinating GAB activities were important to ensure acceptability and feasibility. However, coordinators and the research team, identified feasibility concerns, particularly the poor quality and incompleteness of records from the Office of Medical Records, which raised ethical considerations regarding the recruitment of guardians. For example, of the 62 guardians reached over the phone, 24 children had died, and in seven cases, the child had not been diagnosed with ALL. This raises ethical considerations regarding recruiting guardians from MNH records. In 2018, a certified cause of death was available for only 10% of expected deaths nationally in Tanzania [63]. Researchers recruiting via medical records in Tanzania, where the death rate for the condition is high, e.g., childhood cancer, should have standard protocols to manage potential ethical considerations.

### 4.1 Methodological Considerations

This study has limitations. We did not interview wider research team members. While not directly involved in public contribution activities, wider research team members reviewed impact logs and discussed suggestions and recommendations for implementation. Some impacts may have been missed by only interviewing coordinators and GAB members, e.g., there may have been cultural impacts particularly salient to non‐Tanzanian research team members. Our own preconceptions about the benefits of public contribution may bias results. To mitigate this risk, interviews were conducted by interviewers not involved in public contribution activities, and impact logs and interviews were analyzed by a research team member not involved in public contribution activities. We used impact logs and transformed this qualitative data into quantitative data [33] to calculate the percentage implemented. Impact logs are not standardized quantitative instruments with established psychometric properties and do not account for factors such as the relative weight, complexity, or significance of suggestions or recommendations [41]. Other authors have highlighted the absence of validated quantitative instruments for measuring the impact of public contribution and have called for further research in this area [64]. However, by undertaking a mixed‐method analysis, we triangulated impact log data with interview data to enhance credibility and reviewed impact logs with GAB members to support trustworthiness. In addition, using multiple researchers in categorizing and counting entries helped to enhance consistency and reduce researcher bias.

Despite these limitations, there are important strengths. Our recruitment strategy involved attempting to contact all guardians who met the inclusion criteria, resulting in recruiting guardians from a wide geographical area and with different relationships to the child. Recruitment strategies commonly used in public contribution activities, e.g., choosing contributors already known to the research team, have been criticized for excluding underserved groups [52], which may exacerbate existing inequalities. Random sampling methods have been advocated for recruiting public contributors; however, such strategies are seldom employed [65].

An additional strength was using impact logs and interviews to examine the impact of GAB contribution. A recent systematic review of reviews on the impact of public contribution found 84% of papers failed to report how they measured reported outcomes, or reported outcomes were based on authors' reflections [46]. Furthermore, we used a mixed‐methods approach, integrating transformed impact log data with recruitment data and interview findings using a side‐by‐side comparison table [33, 34]. Through data triangulation, we generated meta‐inferences to demonstrate when and how datasets were convergent, complementary, expanded upon each other, or were silent in relation to key topics arising across datasets [35]. We clearly and transparently illustrated the value of including both qualitative and quantitative data in terms of them offering both convergent and different insights.

## 5 Conclusions

Our findings suggest GAB members' contribution was acceptable and feasible and had several impacts. Impacts on the research were demonstrated by 79% suggestions and recommendations being implemented and were complemented or expanded upon by qualitative findings. Personal impacts on GAB members include building relationships, increasing confidence and empowerment, acquiring knowledge, and motivating caregiving. By demonstrating the acceptability and feasibility of embedding public contribution into a Tanzanian research project, we hope other researchers in Tanzania will consider public contribution activities in their own research.

We will continue to embed public contribution into the GuardiansCan project [16], record public contribution activities and perceived impacts in impact logs, calculate implementation rates, and conduct interviews with GAB members and coordinators at the end of the project [16]. We will report end‐of‐study results upon project completion. Our ongoing mixed‐method examination of public contribution activities throughout the GuardiansCan project will provide additional guidance on how to involve public contributors in research in a meaningful way in a long‐term research project. Long‐term contribution facilitates movements beyond reportedly tokenistic public contribution activities in both LMIC and HIC settings. Furthermore, we hope public contribution can facilitate more equitable research partnerships between LMICs and HICs. In fields such as pediatric oncology, where the emotional and psychological toll on families is immense, public contribution may be particularly valuable to ensure research addresses real‐world concerns and leads to outcomes with direct, practical benefits.

## Author Contributions

**Faraja Chiwanga:** validation, formal analysis, investigation, data curation, writing – original draft, review and editing, visualization; **Guardian Advisory Board:** methodology, formal analysis; **Ruchius Philbert:** data curation, investigation, project administration, writing – review and editing; **David A. Richards:** methodology, writing – review and editing; **Abla Sami:** formal analysis, writing‐review and editing, visualization; **Holly V. R. Sugg:** methodology, formal analysis, writing‐review and editing, visualization; **Ida Österman Menander:** investigation, formal analysis, writing‐review and editing; **Joanne Woodford:** methodology, formal analysis, writing – original draft, writing – review and editing, supervision; **Louise von Essen:** conceptualization, methodology, formal analysis, resources, writing – original draft, writing – review and editing, supervision, project administration, funding acquisition.

## Ethics Statement

This study was approved by the Regional Ethical Review Board in Uppsala, Sweden (Dnr: Dnr 2023‐01381‐01); MNH (MNH/IRB/VOL.1/2023/080); and National Institute for Medical Research (NIMR) (NIMR/HQ/R.8a/Vol.IX/4544), and was conducted in accordance with the Helsinki Declaration, ensuring the welfare and rights of all participants and Good Clinical Practice guidelines.

## Consent

The authors have nothing to report.

## Conflicts of Interest

The authors declare no conflicts of interest.

## Supporting information


**Appendix S1:** Guidance for Reporting Involvement of Patients and the Public Checklist (GRIPP2).

**Appendix S2:** Standards for Reporting Qualitative Research (SRQR).

**Appendix S3:** Personalized name tags, branded notebooks, and carrying bags, and the GAB members.

**Appendix S4:** Plain language summaries in Kiswahili and English.

**Appendix S5:** Suggestions and recommendations made by the Guardians Advisory Board (GAB) to inform the GAB, Study II within the Project (Study II), and the wider GuardiansCan project.

## Data Availability

The data that support the findings of this study are available on request from the corresponding author. The data are not publicly available due to privacy or ethical restrictions.
