# Supplementary material for: “Nothing for Us Without Us”: A Mixed Methods Study Examining the Acceptability, Feasibility, and Impact of Involving Guardians of Children With Acute Lymphoblastic Leukemia in Tanzania as Public Contributors
Source: Cancer Med. 2026 Mar 2;15(3):e71685. doi: 10.1002/cam4.71685 (PMC12953055; doi:10.1002/cam4.71685)
Supplement: Supplementary file 3 — Appendix S3: Personalized name tags, branded notebooks, and carrying bags, and the GAB members. [file CAM4-15-e71685-s001.docx]

**Appendix S3:** Personalized name tags, branded notebooks, and carrying bags, and the GAB members


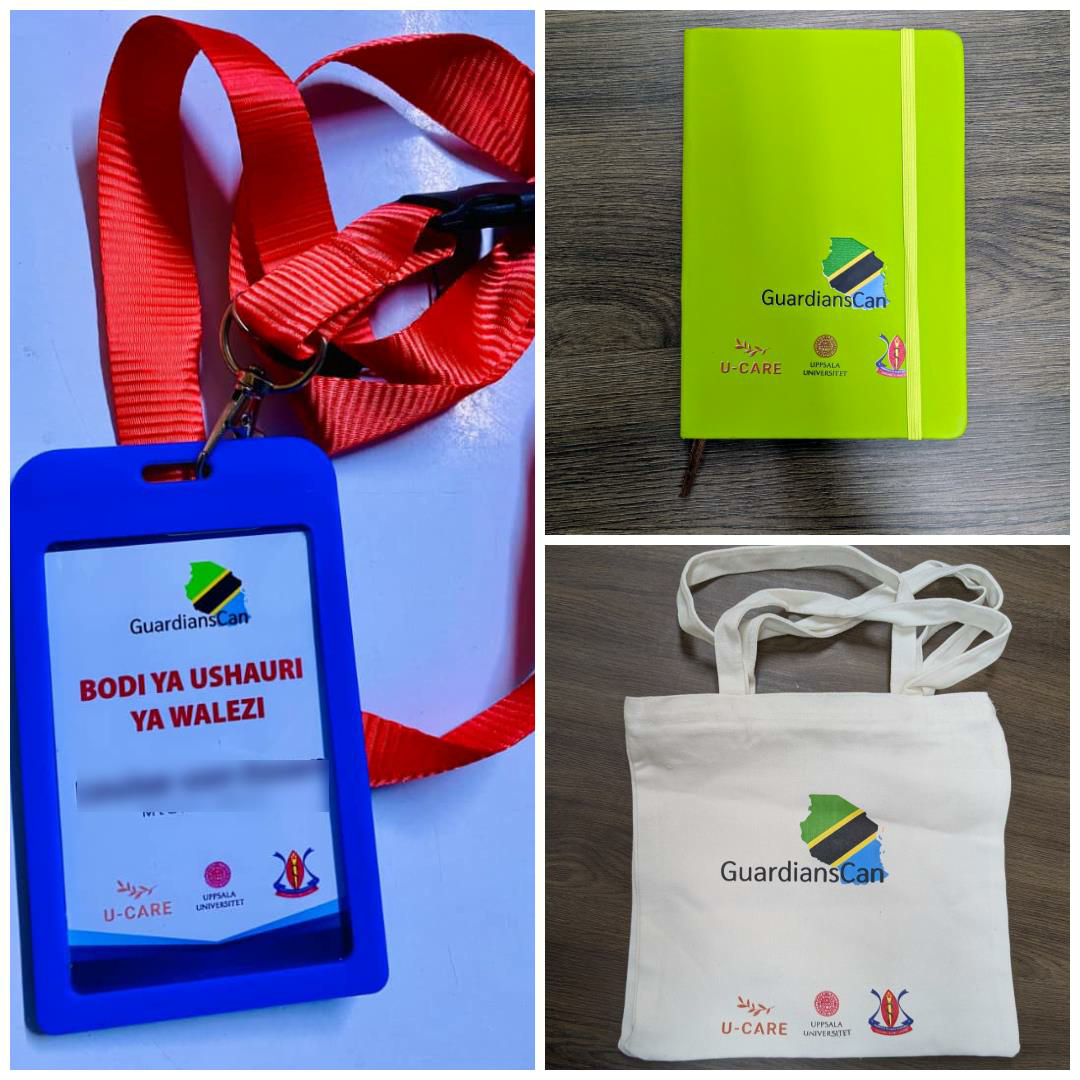


**Figure 1**: Personalized name tags, branded notebooks, and carrying bags


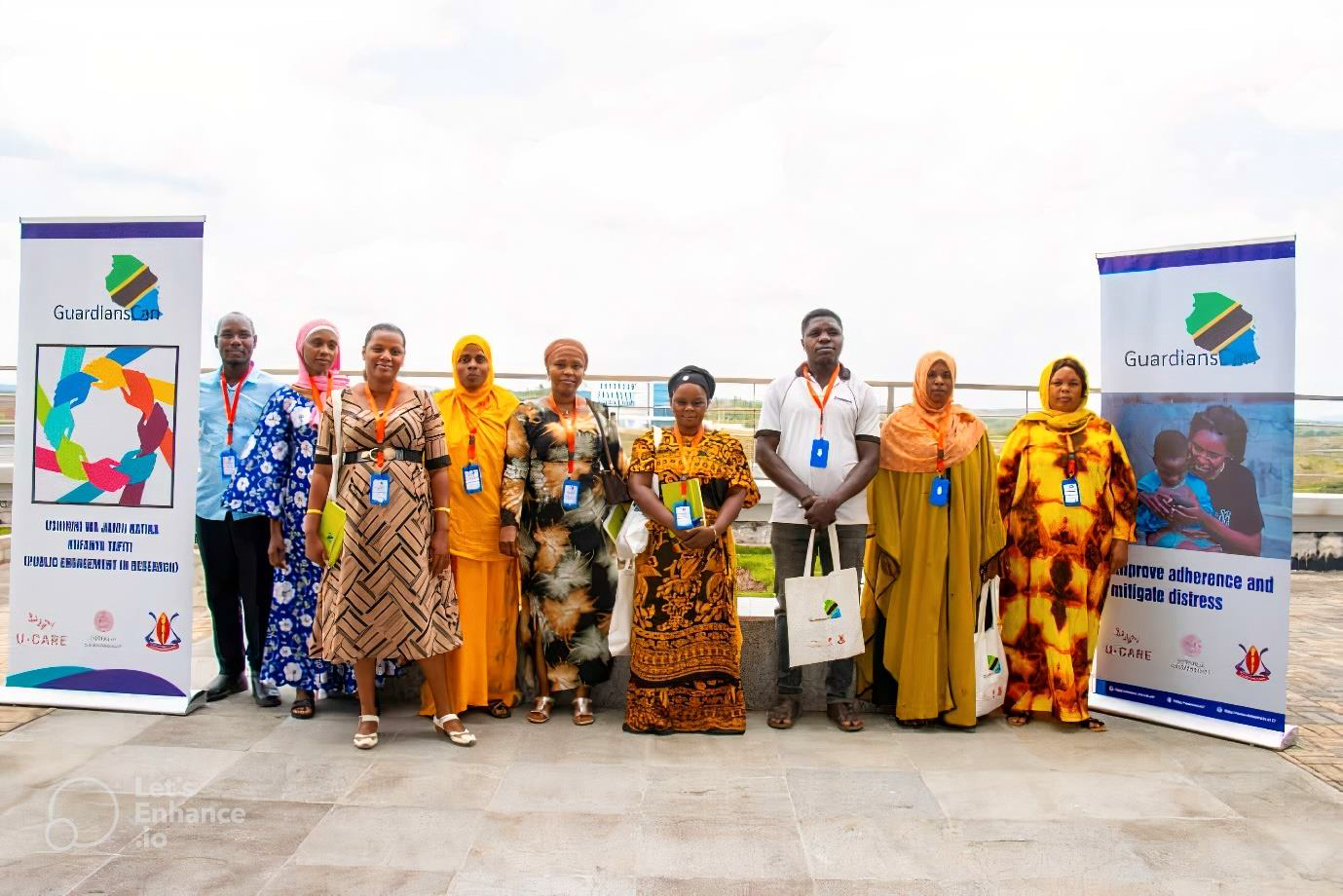


**Figure 2**: Members of the Guardian Advisory Board

*Verbal permission was obtained from the GAB members to use their photograph in this manuscript* *and for other dissemination activities related to the project.*
