# Supplementary material for: “Nothing for Us Without Us”: A Mixed Methods Study Examining the Acceptability, Feasibility, and Impact of Involving Guardians of Children With Acute Lymphoblastic Leukemia in Tanzania as Public Contributors
Source: Cancer Med. 2026 Mar 2;15(3):e71685. doi: 10.1002/cam4.71685 (PMC12953055; doi:10.1002/cam4.71685)
Supplement: Supplementary file 4 — Appendix S4: Plain language summaries in Kiswahili and English. [file CAM4-15-e71685-s002.docx]

**Supplementary Appendix S4**. Plain language summaries in Kiswahili and English

**Plain Language Summary - Kiswahili**

**Utangulizi**

Watoto wengi wenye saratani ya damu hupoteza maisha kutokana na kukosa tiba sahihi ya ugonjwa huu. Mradi wa utafiti wa Guardianscan unalenga walezi wa Watoto wenye saratani ya damu, kuwaelimisha, kuboresha mawasiliano, msaada wa kisaikolojia ya walengwa na kukabilinana na ufuatiliaji hafifu wa matibabu kwa kutumia simu za mkononi.

Utafiti huu umefanywa na walezi wa watoto wenye saratani ya damu na watoa huduma za afya kutoka Hospitali ya Taifa Muhimbili, Tanzania na Chuo Kikuu cha Uppsala, Sweden. Mradi huu umeleta matokeo chanya na ya kuridhisha.

**Lengo la mradi**

Sehemu ya kwanza ya mradi ilihusisha kuwashirikisha walezi/wazazi kama watafiti jamii, katika mradi wa utafiti wa GuardianCan na kupata maoni ya walezi na waratibu wa mradi kufahamu endapo ushiriki wa walezi kama watafiti jamii unawezekana, unakubalika na unaleta tija kwa walezi wenyewe na kwenye mradi.

**Matokeo**

Walezi /wazazi walikubali kushiriki katika mradi kwa hiari kwa sababu waliheshimiwa na kupata uzoefu na hisia ya kuwa sehemu ya jamii. Pia walipata hamasa ya kuwasaidia wengine kihisia, kuondoa hofu na huzuni. Utafiti unaweza kuwa na changamoto mbalimbali ikiwemo hofu, msongo wa kihisia, usafiri mawasiliano na masuala ya familia na tamaduni.

**Hitimisho**

Mradi huu ni muhimu sana kwani una uwezo wa kupunguza vifo vinavyotokana na saratani ya damu kwa watoto kwa kuwa walezi/wazazi wataelimishwa jinsi ya kuwasaidia watoto wao wagonjwa kwa haraka. Ushirikishwaji wa walezi wa watoto utaboresha utekelezaji wa utafiti, hata hivyo ili mradi ufanikiwe, maandalizi mazuri ni ya lazima—kama vile mawasiliano, mazingira salama na usafiri. Hivyo basi, tunaomba Serikali, Wizara ya Afya, na wadau wengine waunge mkono miradi ya utafiti inayowashirikisha wana jamii.

**Plain Language Summary - English**

**Background**
Many children with leukemia lose their lives due to lack of proper treatment for the disease. The GuardiansCan research project targets guardians of children with leukemia—aiming to educate them, improve communication, provide psychological support to affected families, and address poor treatment adherence through the use of mobile phones.

This study was conducted by guardians of children with leukemia and healthcare providers from Muhimbili National Hospital, Tanzania, and Uppsala University, Sweden. The project has yielded positive and satisfactory results.

**Project aim**

The first part of the project involved engaging guardians/parents as community researchers in the GuardiansCan research project. It also sought feedback from guardians and project coordinators to understand whether involving guardians as community researchers is feasible, acceptable, and beneficial both for the guardians themselves and for the project.

**Findings**
Guardians/parents voluntarily agreed to participate in the project because they felt respected and experienced a sense of belonging within the community. They were also motivated to help others emotionally, alleviating fear and sadness. Research can present various challenges, including fear, emotional stress, transportation, communication, and issues related to family and culture.

**Conclusion**
This project is very important as it has the potential to reduce deaths due to childhood leukemia because guardians/parents will be educated on how to support their sick children promptly. Involving guardians of children will improve the research implementation, however in order for it to succeed, proper preparations are necessary, such as communication, a safe environment, and transportation. Therefore, we call upon the Government, the Ministry of Health, and other stakeholders to support such research projects that involve the public.
