# Supplementary material for: “Nothing for Us Without Us”: A Mixed Methods Study Examining the Acceptability, Feasibility, and Impact of Involving Guardians of Children With Acute Lymphoblastic Leukemia in Tanzania as Public Contributors
Source: Cancer Med. 2026 Mar 2;15(3):e71685. doi: 10.1002/cam4.71685 (PMC12953055; doi:10.1002/cam4.71685)
Supplement: Supplementary file 5 — Appendix S5: Suggestions and recommendations made by the Guardians Advisory Board (GAB) to inform the GAB, Study II within the Project (Study II), and the wider GuardiansCan project. [file CAM4-15-e71685-s004.docx]

**Appendix S5**. Suggestions and recommendations made by the Guardians Advisory Board (GAB) to inform the GAB, Study II within the Project (Study II), and the wider GuardiansCan project

| **Topics** | **Suggestions** | **Rationale for suggestions** | **Implemented in** | | | | | **Rationale for non-implementation** |
| --- | --- | --- | --- | --- | --- | --- | --- | --- |
|  |  |  | **GAB** | **Study II** | | **Guardians-Can Project** | |  |
| **Workshop 1: Introduction to the GuardiansCan project and the GAB** | | | | | | | | |
| Introduction to the GAB, the project, and public contribution to research | Not applicable. |  |  | |  | |  |  |
| GAB members’ expectations | Not applicable. |  |  | |  | |  |  |
| GAB Structure (e.g., frequency, and breaks); and setting for GAB workshops | Workshop duration to be 4 hours. |  | Yes | |  | |  |  |
|  | Workshops to include a break for breakfast. |  | Yes | |  | |  |  |
|  | Workshops to end before lunch. |  | Yes | |  | |  |  |
|  | Workshops to be held at MNH-Mloganzila. |  | Yes | |  | |  |  |
|  | Workshop room should have good-quality chairs. | Existing chairs in room are uncomfortable and hurt GAB member’s backs. | Yes | |  | |  |  |
|  | Workshop schedule planned until the end of 2024 is okay. |  | Yes | |  | |  |  |
|  | Workshops should not overlap with days guardians visit the paediatric oncology clinic, i.e., Tuesdays and Fridays. Thursday is a good day. |  | Yes | |  | |  |  |
|  | GAB members would like to continue staying together in the hotel. |  | Yes | |  | |  |  |
|  | GAB members should have project t-shirts. | This will make it easier for guardians to be recognized. | Yes | |  | |  |  |
| Communication plan for the GuardiansCan project and forthcoming results | Use phone calls and send an SMS if the call is not answered. |  | Yes | |  | |  |  |
|  | Contact GAB members two weeks in advance to remind them about the next workshop and again five days before the workshop. |  | Yes | |  | |  |  |
| Any other business | Help GAB members obtain permission from partners and/or their work to participate in the workshops. |  | No | |  | |  | Does not comply with research ethics code of conduct, i.e., to respect human dignity, privacy, and autonomy. |
|  | Provide help to guardians who have a low income so that they can send their children for treatment. |  |  | |  | | No | Outside of scope of this project. |
|  | Recruit more GAB members via existing GAB members as they know each other. |  | No | |  | |  | Risk of selection bias. |
|  | Recruit more GAB members via local clinics that provide care to children with cancer near to where the children live. |  | No | |  | |  | Risk of selection bias and clinician gatekeeping. |
|  | Develop a mHealth intervention that does not rely on smartphones. |  |  | |  | | Yes |  |
|  | Advise hospitals to provide psychological services for children and guardians of children with cancer. |  |  | |  | | No | Outside of scope of this project. |
|  | Provide guardians of children with cancer regular seminars to educate them on how to take care of the children. |  |  | |  | | No | Outside of scope of this project. |
| **Workshop 2: Understanding and initial planning of Study II** | | | | | | | | |
| Introduction to photovoice technique, FGD, and individual interview and discussion of the relevance of using these methods | All three methods are relevant but in order of preference, the list is:   1. FGD 2. Individual interview 3. Photovoice technique.   Use FGDs in Study II. | FGDs are preferred as they save time, allow you to understand common thoughts, collect a lot of information at the same time, and may facilitate for those who are afraid to speak/open up.  Individual interviews may be better for those afraid to speak in front of a group.  The photovoice technique may be difficult to understand and use. It requires more creativity and the ability to translate the photo into what you want to express. More training may be needed. However, it could be beneficial for those who find it difficult to express themselves. |  | | Yes | |  |  |
| Recruitment of study participants, e.g., methods and procedures to use and regions to recruit from | Add an additional region, such as Tanga which is not too far from Dar es Salaam. |  |  | | Yes | |  |  |
|  | Take into consideration that Zanzibar has different regions which are not necessarily similar demographically. |  |  | | Yes | |  |  |
|  | Continue with the same recruitment procedure, i.e., a doctor makes the call. | Even spouses at home will trust the phone call when they know a doctor has called. |  | | Yes | |  |  |
|  | Recruit study participants via GAB members calling guardians that they know. |  |  | | No | |  | Risk of selection bias. |
|  | Make phone calls between 4 pm and 8 pm. | This is the time people are at home. |  | | Yes  *Note*: these times are prioritized, we will also call at other times. | |  |  |
|  | Make phone calls up to 11 pm. | Some people come back home late. |  | | No | |  | Calling after 9 pm is too late for the research team. |
|  | Make up to three phone calls up to three times. |  |  | | No | |  | Given our experience with GAB recruitment, flexibility is needed to make more phone call attempts is needed. Up to five calls will be made over two weeks. |
|  | Make phone calls over two weeks. |  |  | | Yes | |  |  |
|  | Send an SMS every time you call informing them that you are a doctor from MNH, and ask guardians to inform you when they are available so you can call them. | If someone has a child who has been treated by MNH, they will call back. |  | | No | |  | Given our experience with GAB recruitment, we do not have reliable contact details, and therefore, it is not possible to send an SMS before we have confirmed the guardian's phone number. Once we have made contact with a guardian and confirmed it is the correct person, SMS communication can be used. |
|  | Ask guardians about the condition of the child first. | If the child has passed away, there is no need to continue the recruitment phone call as was done when recruiting the GAB. |  | | Yes | |  |  |
|  | Recruit male study participants via GAB members calling guardians that they know. | It is difficult to find men because they often do not stay with children and may not be listed on hospital records. |  | | No | |  | Risk of selection bias. |
|  | Ensure that recruitment from Zanzibar considers the island’s five regions: North Unguja, South Unguja, Urban West Unguja, South Pemba, and North Pemba. | Recruiting from only one region in Zanzibar will not be representative of the region as a whole.  Most areas in Unguja have no cultural differences, but Pemba includes areas that are very rural, which differentiates it from other regions in Zanzibar. |  | | Yes | |  |  |
| Background questions | No need to ask questions about religion, tribe, or type of phone. Other questions are appropriate. | Tanzania has no culture for people to want to know each other by religion or tribe.  It may be offensive to ask questions about someone's phone. |  | | Yes | |  |  |
| Frequency and setting of FGDs and whether GAB should facilitate FGDs | Hold FGDs at MNH-Mloganzila. |  |  | | Yes | |  |  |
|  | Hold two or three FGDs. |  |  | | Yes  *Note*: Three FGDs will be planned, however, we may add additional FGDs if we do not get enough data. | |  |  |
|  | Hold FGDs once a month. The first two discussions can be held over two days. |  |  | | Yes | |  |  |
|  | Hold an introductory and “ice-breaking” meeting on the first day and hold the first FGD the day after. |  |  | | No | |  |  |
|  | Hold FGDs for between one and two hours. | More than two hours can be exhausting for the participants. |  | | No | |  | One or two hours is not enough time, especially given that breaks may be needed and there can be interruptions. We will need two hours of data collection time, and breaks will be planned in addition to these two hours. |
|  | Guardians should not facilitate FGDs. | The advantage is guardians will learn how to conduct FGDs. The disadvantage is that the guardians know each other and may not be open to discussing some challenges in front of those they know. |  | | Yes | |  |  |
| Additional topics (i.e., topics added to the original Workshop 2 agenda after Workshop 1 by the research team informed by Workshop 1 discussions). Additional topics included the availability of mobile phones and motivation to participate in research | Develop an intervention suitable for all types of phones, such as Unstructured Supplementary Service Data (USSD) and mass messages (Bulk SMS services). | Many people do not have smartphones. |  | |  | | Yes  *Note*: We will co-design an SMS text message intervention with the GAB. |  |
|  | Explain the project in detail, including its potential benefits when inviting guardians. |  |  | | Yes | |  |  |
|  | Develop an intervention that allows guardians with sick children to talk to other guardians to give advice and encourage them. |  |  | |  | | To consider | We will make decisions concerning the type of intervention during co-design with the GAB. |
|  | There should be a possibility for guardians of children who have recovered to motivate other guardians with their testimonies. |  |  | |  | | To consider | We will make decisions concerning the type of intervention during co-design with the GAB. |
| Any other business | Include women and men in FGDs. | There are no challenges with women and men being together in FGDs. |  | | Yes | |  |  |
|  | Do not include spouses of GAB members in the GAB. | The partners of two female GAB members asked them whether they could join the GAB. They do not want their partners to join as they will not be free to express their feelings. | Yes | |  | |  |  |
| Information on topics to be covered in Workshop 3 | Make a travel plan involving two days and sleeping in their district to catch the bus or boat the morning of the day before GAB workshops. | The two guardians who travel from far places arrive late at night and are tired. Travelling for two days would be less tiring. | Yes | |  | |  |  |
| **Workshop 3: Further planning of Study II** | | | | | | | | |
| Presentation and discussion of notes in Workshop 2 impact log and decisions made by the research team on discussed aspects | Make as many calls as possible within a period of two weeks and at different hours if the phone is “not available”. | If the phone is not available, there is no reason to set a limit of five times because there is no disturbance to the person being called. |  | | Yes | |  |  |
|  | Add questions about the age of the child and the gender of the child to the Background Questions. |  |  | | Yes | |  |  |
| Written information and consent forms | No need to add to or amend the participant information sheet. | The participant information sheet is clear and correct. |  | | Yes | |  |  |
|  | No need to add to or amend the consent form. | The consent form is clear and correct. |  | | Yes | |  |  |
| Topic guides for FGDs | Part 5: Obstacles and enablers  Modify question (d) so that it can be understood by guardians. It will now read: What would help you to trust the information or education you would receive through mobile technology? |  |  | | Yes  *Note*: Old FGD topic guide used in workshop, the research team had already amended this question. | |  |  |
|  | Part 7: Expectations Modify question (b) so that it can be understood by guardians. It will now read: In what way you would like the mobile technology or program to work? (e.g., WhatsApp, short message, calling through a free number). |  |  | | Yes  *Note*: Old FGD topic guide used in workshop, the research team had already amended this question. | |  |  |
| Additional topics (i.e., topics added to the original Workshop 3 agenda after Workshop 2 by the research team informed by Workshop 2 discussions). Additional topics included exploring whether GAB members would be willing to try the photovoice technique in the future, asking if they would be interested in taking part in an upcoming public contribution training, and informing them about the planned involvement of HCPs in Study II, and asking for their input on questions to include in the FGDs with HCPs. | Add a question to the FGD topic guide for HCPs:  What do you do as HCPs to help guardians with psychological difficulties? |  |  | | Yes | |  |  |
| Any other business | No suggestions or recommendations. |  |  | |  | |  |  |
| Information on topics to be covered in Workshop 4 | No suggestions or recommendations. |  |  | |  | |  |  |
| **Workshop 4: Final planning of Study II** | | | | | | | | |
| Presentation and discussion of notes in Workshop 3 impact log and decisions made by the research team on discussed aspects | No suggestions or recommendations. |  |  | |  | |  |  |
| Recruitment of HCPs in Study II | Recruit doctors and nurses as planned and add pharmacists, social workers, and psychologists. |  |  | | Yes | |  |  |
|  | No need to add to or amend the FGD guide for HCPs. |  |  | | Yes | |  |  |
| Additional topics (i.e., topics added to the original Workshop 4 agenda after Workshop 3 by the research team informed by Workshop 3 discussions). Additional topics included a second review of written information, consent forms, and topic guides for FGDs | No need to add to or amend the participant information sheet. | • Understandable language. • No words need to be changed. • The purpose of the study is understandable. • Things that the participants must do are understandable. • The flow of questions is good and understandable. • The order of the questions is good. |  | | Yes | |  |  |
|  | No need to add to or amend the consent form. | • Understandable language.  • No words need to be changed.  • The flow of questions is good and understandable.  • The order of the questions is good. |  | | Yes | |  |  |
|  | No need to add to or amend the background questionnaire. | • Understandable language.  • No words need to be changed.  • The flow of questions is good and understandable.  • The order of the questions is good.  The questions asked make sense. |  | | Yes | |  |  |
|  | No need to add to or amend the FGD guide. | • Understandable language.  • No words need to be changed.  • The flow of questions is good and understandable.  • The order of the questions is good.  • The order of questions makes sense. |  | | Yes | |  |  |
| Ethical considerations, such as privacy, identity protection, and use of sensitive information | Implement communication of results to study participants. |  |  | | Yes | |  |  |
|  | Do not involve GAB members in FGDs. | Other guardians may be worried about sharing their sensitive information. |  | | Yes | |  |  |
|  | Include possible risks that guardians may be reminded of difficult feelings. |  |  | | Yes | |  |  |
|  | Include that a possible risk may include arriving at Dar es Saleem at night. Mitigate this risk with good communication with the research team about the time when arriving and assigning a driver to collect guardians. | GAB members have not considered themselves in any danger when arriving at night because there has always been good communication between public contribution coordinators and the driver assigned to pick them up. |  | | Yes | |  |  |
| Any other business | Inform GAB members that they will be interviewed by phone in advance so that they can organize themselves and be present in an area with a mobile network. |  | Yes | |  | |  |  |
| Discussion about logistics for individual interviews with GAB members and information on the next steps, e.g., finalizing study II protocol and submission for ethical approval | No suggestions or recommendations. |  |  | |  | |  |  |

*Notes.* GAB = Guardians Advisory Boards; MNH = Muhimbili National Hospital; FGD = focus group discussions; HCP = healthcare professional.
